# Differential influences of mpox illness representation on sexual behaviors among gay, bisexual, and other men who have sex with men in Beijing and Hong Kong

**DOI:** 10.1186/s12879-026-13062-7

**Published:** 2026-03-13

**Authors:** Lijuan Wang, Siyu Chen, Yanjie Gao, Fuk-yuen Yu, Yuan Fang, Haifeng Ding, Xinge Li, Yingjie Liu, Zihuang Chen, Zhennan Li, Phoenix K. H. Mo, Zixin Wang

**Affiliations:** 1 Beijing Chaoyang District Center for Disease Control and Prevention, Beijing, China; 2 Centre for Health Behavours Research, JC School of Public Health and Primary Care, The Chinese University of Hong Kong, Hong Kong, China; 3 Department of Health and Physical Education, The Education University of Hong Kong, Hong Kong, China; 4 Danlan Goodness, Beijing, China; 5 Hongshiliu Goodness, Beijing, China

**Keywords:** Mpox, Illness representations, Sexual behaviors, Gay, bisexual, and other men who have sex with men, China

## Abstract

**Background:**

Mpox perceptions may influence the onset of sexual behaviors (e.g., condomless anal sex [CAS] with men, number of male sex partners, and sexualized drug use) among gay, bisexual, and other men who have sex with men (GBMSM). However, no studies have examined how mpox illness representations relate to sexual behaviors. This study examined the associations between mpox illness representations and presence of sexual behaviors among GBMSM in Beijing and Hong Kong.

**Methods:**

This secondary analysis of a cross-sectional survey conducted between November 2023 and March 2024 among GBMSM in Beijing and Hong Kong. Eligible participants were Chinese males aged ≥ 18 years who had anal sex with at least one man in the past six months. Participants were recruited through venue-based outreach, online platforms, and peer referrals, and completed a structured telephone interview. Multivariable logistic and negative binomial regression models assessed the associations between mpox illness representations and sexual behaviors.

**Results:**

A total of 524 GBMSM in Beijing and 613 in Hong Kong participated (response rates: 87% and 86%). After adjustment, GBMSM in Beijing perceived more severe consequences, longer expected illness timeline, greater personal control, more concern, and better understanding related to mpox than their counterparts in Hong Kong. In Beijing, better understanding of mpox (coherence) was associated with lower odds of CAS with men (adjusted odds ratio = 0.92, 95% CI: 0.87–0.98), while longer expected illness timeline (timeline) (incidence rate ratio [IRR] = 0.96, 95% CI: 0.93–1.00), anticipating more severe symptoms if infected (identity) (IRR = 0.94, 95% CI: 0.90–0.97) and stronger negative emotions (emotion) (IRR = 0.94, 95% CI: 0.90–0.98) were associated with fewer male sex partners. In contrast, greater personal control (IRR = 1.06, 95% CI: 1.02–1.10) was associated with higher number of male sex partners in Beijing.

**Conclusions:**

Associations between mpox-related illness perceptions and sexual behaviors were observed between GBMSM in Beijing but were not detected in Hong Kong. Future studies may explore whether modifying mpox-related illness representation is helpful to promote safe sexual behaviors among GBMSM in Beijing.

**Trial registration:**

Not applicable.

**Supplementary Information:**

The online version contains supplementary material available at 10.1186/s12879-026-13062-7.

## Background

The mpox outbreak has become a major global public health concern. Mpox is a zoonotic viral disease caused by the monkeypox virus [1]. Since May 2022, human-to-human transmission has been reported worldwide [2]. Most cases globally and locally have been associated with close physical or sexual contact, particularly among gay, bisexual, and other men who have sex with men (GBMSM) [3–5]. Mainland China reported its first imported mpox case in September 2022 and there were more than 2,000 confirmed cases in 2024 [6]. In Hong Kong, a special administrative region of China, 81 mpox cases were recorded since 2022 [7]. Both mainland China and Hong Kong implemented surveillance, isolation, contact tracing, and public education to control transmission [4, 8]. However, mpox vaccination was available in Hong Kong but not in mainland China (including Beijing). The fully-subsidized JYNNEOS vaccine was offered to high-risk groups in Hong Kong, including GBMSM [8–10].

Sexual behaviors play important roles in mpox transmission among GBMSM [11, 12]. In mainland China and Hong Kong, previous surveys reported high prevalence of condomless anal sex (CAS) (53% and 52.4%), multiple male sex partners (34% and 29.4%), and sexualized drug use (SDU) (28.4% and 9.3%) [13–15]. These sexual behaviors are shaped by factors such as age, education, relationship status, online partner-seeking, the use of HIV prevention services (pre-exposure prophylaxis [PrEP] and HIV/sexually transmitted infections [STIs] testing), and perceptions related to HIV (e.g., risk perceptions) [16, 17]. In addition to HIV, perceptions related to other STIs (e.g., HPV, genital warts) could also influence the occurrence of sexual behaviors among GBMSM [18]. It is possible that perceptions related to mpox would also influence the onset of these sexual behaviors. HIV prevention workers have been advocating for integrated HIV and STI prevention strategies. Understanding how mpox-related perceptions would affect sexual behaviors among GBMSM is therefore important for guiding such prevention strategies. To our knowledge, there are no reported studies investigating the relationship between mpox-related perceptions and sexual behaviors among GBMSM.

Illness representations describe how individuals perceive an illness, including its causes, consequences, controllability, and emotional impact. These perceptions shape the responses to health threats (e.g., mpox) and preventive behaviors. According to Leventhal’s Common-Sense Model of Self-Regulation, illness representations consist of multiple perception domains, such as identity (symptoms and labeling), cause, timeline, consequences, personal and treatment control, coherence (overall understanding of the illness), and emotional responses to the illness [19]. Previous studies have examined how individual illness representation domain would affect health-related behaviors, including influenza [20] and pneumococcal vaccination [21], hepatitis C testing [22], and cancer screening [23]. A recent study in Hong Kong found that illness representations related to mpox were associated with mpox vaccine uptake among GBMSM [5]. However, no research has examined whether mpox illness representations are associated with sexual behaviors. As illness representations are modifiable through interventions [24], understanding GBMSM’s beliefs about mpox may help inform strategies to promote safer sexual behaviors.

To address the knowledge gaps, we conducted a cross-sectional study examining the associations between mpox illness representations and presence of sexual behaviors (e.g., CAS with men, number of male sex partners, and SDU) among GBMSM in Beijing and Hong Kong. We hypothesized that the level of mpox illness representations and their relationships with sexual behaviors would be different between GBMSM in Beijing and Hong Kong.

## Methods

### Study design

This was a secondary analysis of a cross-sectional survey conducted between November 2023 and March 2024 among GBMSM in Beijing and Hong Kong. Beijing locates in northern China, which is the capital city of People’s Republic of China and one of the most developed cities in the country. Hong Kong locates in southern China and is a special administrative region of the People’s Republic of China. The population size was 21.8 and 7.5 million in Beijing and Hong Kong at the time of the study. Both Beijing and Hong Kong have large GBMSM communities and have implemented surveillance, isolation, contact tracing, and public education to control mpox transmission [4, 8]. However, there are two major differences related to mpox between the study sites. As compared to Hong Kong, the number of reported mpox cases was higher in Beijing (258 vs. 53) during the study period [4, 25]. In addition, mpox vaccination is available in Hong Kong but not in Beijing.

### Participants and data collection

Participants were eligible if they: (1) were Chinese males aged ≥ 18 years, and (2) had anal intercourse with at least one man in the past six months. Recruitment procedures were the same in both cities [26, 27]. Trained fieldworkers approached potential participants in gay bars during weekdays and weekends with venue approval. Online recruitment was also conducted through gay websites, social media, and partner-seeking apps. In Hong Kong, advertisements were posted on gay websites, Facebook, and Grindr. Facebook is the most widely used social media platform among local GBMSM [28], and Grindr is the most popular gay dating app [29]. In mainland China, these platforms are unavailable; therefore, recruitment used Weibo and WeChat (similar functions to Facebook) and Blued, the most widely used gay dating app in China [30]. Interested individuals contacted the research team by private message, telephone, WhatsApp/WeChat, or email. Peer referrals and collaboration with community-based organizations further supplemented recruitment. After eligibility screening, participants were informed of the study and confidentiality. Written informed consent was waived by the Survey and Behavioral Research Ethics Committee of the Chinese University of Hong Kong (reference no. SBRE-22-0488) to maintain anonymity. Instead, verbal informed consent was obtained, and fieldworkers signed a form pledged that the participants had been fully informed about the study. Similar practice was used in numerous published studies targeting GBMSM [31, 32]. Structured telephone interviews were conducted in Mandarin (Beijing) and Cantonese (Hong Kong).

Questionnaires were administered verbally in simplified Chinese (Beijing) and traditional Chinese (Hong Kong), with identical content to ensure comparability. Trained interviewers read each question and response option aloud. Our team has extensive experience conducting telephone surveys with GBMSM [26, 27, 33]. Compared with online self-administered surveys, telephone interviews generally had higher completion rates and allow clarification of complex questions. Each interview lasted about 30 min. Upon completion, participants received a HK$50 (US$6.4) supermarket coupon in Hong Kong or a ¥20 (US$3.0) electronic coupon in Beijing, consistent with previous studies [32–34].

### Measurements

#### Development of the questionnaire

A panel was developed to design the questionnaire, including public health researchers, behavioral health scientists, health psychologists, staff from community-based organizations, representatives from the Center for Disease Control and Prevention, and local GBMSM. A pilot test with 20 GBMSM (10 in each city) assessed clarity and acceptability. Participants reported that the questions were easy to understand and the length was acceptable. Based on their feedback, the panel finalized the questionnaire for the survey. These 20 pilot participants were not included in the target sample size.

#### Background characteristics

Participants reported their sociodemographic characteristics, including age, relationship status, education level, employment status, monthly personal income, sexual orientation and HIV sero-status. Additional questions assessed history of SARS-CoV-2 infection (confirmed by rapid antigen test and/or nucleic acid amplification test), COVID-19 vaccination status, and mpox vaccination (yes/no). Participants also reported use of HIV testing, PrEP, STI testing, and other HIV-related services (e.g., receiving condoms, lubricants, educational materials, seminars/workshops).

#### Dependent variables: sexual behaviors in the past six months

Participants reported the number of regular and non-regular male sex partners with anal sex, CAS with different type of male sex partners, and engagement in SDU in the past six months. Regular male sex partners (RP) were defined as their stable boyfriends or lovers, while non-regular male sex partners (NRP) were defined as casual sex partners or male sex workers [33]. SDU was defined as the use of psychoactive substances before or during sex, including ketamine, methamphetamine, cocaine, cannabis, ecstasy, Dormicum, Halcion, Erimin-5, non-prescription hypnotics, heroin, cough suppressants (used recreationally rather than for treating cough), amyl nitrite (poppers), gamma-hydroxybutyrate or gamma-butyrolactone, 5-methoxy-N, N-diisopropyltryptamine, and mephedrone [33, 35].

#### Mpox illness representations

The measurement tool was adapted from the validated Chinese version of Brief Illness Perception Questionnaire (BIPQ) [36]. The Chinese version of BIPQ has been used in different population in both Beijing and Hong Kong [37, 38], including GBMSM [22]. We replaced the word “illness” by “mpox” in the validated Chinese version of BIPQ. The Cronbach’s alpha of the adopted tool was 0.84. This method has been applied in previous studies to assess illness representations among individuals at risk of a disease, but who do not have the disease [39, 40]. Each item was rated on a Likert scale ranging from 0 to 10. The domains included consequences (“If you contracted mpox, how much would it affect your life?”), timeline (“If you contracted mpox, how long do you think it would last?”), personal control (“If you contracted mpox, how much control would you feel you have over it?”), treatment control (“If you contracted mpox, how much do you think available treatment could help?”), identity (“If you contracted mpox, how many symptoms would you expect to experience?”), concern (“If you contracted mpox, how concerned would you be?”), coherence (“If you contracted mpox, how well do you feel you understand it?”), and emotional response (“If you contracted mpox, how much would it affect you emotionally?”).

### Statistical analysis

Frequency distributions were calculated for all study variables. Between-city differences in background characteristics were examined using chi-square tests. Between-city differences in CAS with men and SDU, as well as mpox illness perceptions, were assessed using multivariable logistic regression models for binary outcomes and linear regression models for continuous outcomes, adjusting for background characteristics with significant between-city differences. The number of male sex partners with anal intercourse, a count outcome exhibiting substantial overdispersion, was analyzed using negative binomial regression models. CAS with men, SDU, and number of male sex partners with anal intercourse were used as dependent variables. Within each city, multivariable regression models were used to examine the associations between mpox illness representations and each dependent variable. Each model included one independent variable of interest (e.g., a domain of mpox illness perception) and all significant background characteristics. Results from logistic regression models are presented as adjusted odds ratios (AORs) and results from negative binomial regression models are presented as incidence rate ratios (IRRs), all with corresponding 95% confidence intervals (CIs). In addition, sensitivity analyses were conducted among participants without a history of mpox vaccination in both cities to examine whether the observed associations remained consistent among unvaccinated GBMSM across the two settings. Multicollinearities were checked using the variance inflation factor (VIF); variables with a VIF value greater than 5 were re-scaled or removed if theoretically redundant. Model fits of multivariable logistic regression models were assessed using the Hosmer and Lemeshow Goodness-of-fit test. All analyses were conducted in SPSS version 26.0 (IBM Corp., Armonk, NY, USA). A two-sided p-value of less than 0.05 was considered statistically significant.

## Results

### Background characteristics of the participants

The research team approached 602 and 713 eligible GBMSM in Beijing and Hong Kong. Of them, 78 in Beijing and 100 in Hong Kong declined participation, mainly due to a lack of time or other tangible reasons. In total, 524 and 613 participants in Beijing and Hong Kong completed the telephone interviews, respectively. The response rate, which was defined as the number of completed interviews divided by the number of eligible participants being approached, was comparable between Beijing and Hong Kong (87% vs. 86%).

As compared to GBMSM in Beijing, those in Hong Kong were more likely to be older (≥ 35 years old: 43.9% vs. 34.1%, *p* < 0.001), single (82.1% vs. 70.6%, *p* < 0.001), having a monthly personal income at or above the median level of the city (68.8% vs. 56.3%, *p* < 0.001), gay (89.6% vs. 77.3%, *p* < 0.001), and receiving a mpox vaccination (23.3% vs. 1.0%, *p* < 0.001). More participants in Beijing self-reported as HIV positive comparing to their counterparts in Hong Kong (6.1% vs. 1.3%, *p* < 0.001). More participants in Beijing utilized HIV testing (84.7% vs. 46.3%, *p* < 0.001), PrEP (18.3% vs. 14.3%, *p* = 0.03), STI testing (63.7% vs. 38.8%, *p* < 0.001) and other HIV-related services (59.9% vs. 44.5%, *p* < 0.001) than those in Hong Kong (Table 1).

**Table 1:** Background characteristics of GBMSM in Beijing and Hong Kong, China

	Beijing (*n* = 524)	Hong Kong (*n* = 613)	*p* value
	*n* (%)	*n* (%)	
**Socio-demographic characteristics**			
Age group (years)			
18–24	67 (12.8)	72 (11.8)	
25–34	278 (53.1)	271 (44.3)	
35–44	138 (26.3)	171 (27.7)	
45 or above	41 (7.8)	99 (16.2)	< 0.001
Current relationship status			
Currently single	370 (70.6)	503 (82.1)	
Married or cohabited with a man	119 (22.7)	105 (17.1)	
Married or cohabited with a woman	35 (6.7)	5 (0.8)	< 0.001
Highest education level attained			
Senior high or below	69 (13.2)	99 (16.2)	
College or above	455 (86.8)	514 (83.8)	0.16
Having a full-time employment			
Yes	417 (79.6)	474 (77.3)	
No	107 (20.4)	139 (22.7)	0.36
Monthly personal income			
Below median income level ^1^	194 (37.0)	187 (30.5)	
Median income or above	295 (56.3)	422 (68.8)	
Refuse to disclose	35 (6.7)	4 (0.7)	< 0.001
Sexual orientation			
Gay	405 (77.3)	549 (89.6)	
Bisexual	81 (15.5)	59 (9.6)	
Heterosexual	7 (1.3)	2 (0.3)	
Uncertain	31 (5.9)	3 (0.5)	< 0.001
Self-reported to be HIV positive			
No	492 (93.9)	605 (98.7)	
Yes	32 (6.1)	8 (1.3)	< 0.001
History of confirmed SARS-CoV-2 infection			
No	83 (15.8)	109 (17.7)	
Yes	441 (84.2)	504 (82.2)	0.38
Number of doses of COVID-19 vaccination received by the participants			
0	22 (4.2)	19 (3.0)	
1	8 (1.5)	9 (1.5)	
2	89 (17.0)	86 (14.0)	
3	375 (71.6)	419 (68.4)	
4	23 (4.4)	76 (12.4)	
5	7 (1.3)	4 (0.7)	< 0.001
Uptake of mpox vaccination			
No	519 (99.0)	470 (76.7)	
Yes	5 (1.0)	143 (23.3)	< 0.001
**HIV-related services utilization in the past six months**			
Use of any types of HIV testing			
No	80 (15.3)	329 (53.7)	
Yes	444 (84.7)	284 (46.3)	< 0.001
Use of pre-exposure prophylaxis			
No	428 (81.7)	517 (84.3)	
Yes	96 (18.3)	88 (14.3)	0.03
Testing for other sexually transmitted infections			
No	190 (36.3)	375 (61.2)	
Yes	334 (63.7)	238 (38.8)	< 0.001
Use of other HIV-related services			
No	210 (40.1)	340 (55.5)	
Yes	314 (59.9)	273 (44.5)	< 0.001

^1^ Median income of the residents was ¥7,000 (US$980) per month in Beijing and HK$20,000 (US$2,564) per month in Hong Kong

### Differences in sexual behaviors and mpox disease perceptions

After adjusting for background differences, GBMSM in Hong Kong reported a higher prevalence of CAS (56.0% vs. 44.1%, *p* < 0.001) but a lower prevalence of SDU (9.3% vs. 18.3%, *p* < 0.001) than those in Beijing. The mean (standard deviation [SD]) number of male sex partners with anal intercourse was similar between GBMSM in Beijing (4.1 [8.3]) and Hong Kong (3.7 [5.8]; *p* = 0.87) (Table 2).

**Table 2:** Sexual behaviors and mpox disease perceptions

	Beijing (*n* = 524)	Hong Kong (*n* = 613)	Unadjusted *p* value	Adjusted *p* value ^1^
	*n* (%)	*n* (%)		
**Sexual behaviors in the past six months**				
Condomless anal sex with men				
No	293 (55.9)	270 (44.0)		
Yes	231 (44.1)	343 (56.0)	< 0.001	< 0.001
Number of male sex partners with anal intercourse				
Mean (SD)	4.1 (8.3)	3.7 (5.8)	0.34	0.87
Sexualized drug use				
No	428 (81.7)	556 (90.7)		
Yes	96 (18.3)	57 (9.3)	< 0.001	0.07
**Mpox disease perceptions**				
Domains of the Brief Illness Perception Questionnaire (B-IPQ), mean (SD)				
If you contracted mpox, how much would it affect your life (consequences) ^2^	7.9 (2.7)	7.3 (2.2)	< 0.001	< 0.001
If you contracted mpox, how long do you think it would last (timeline) ^3^	7.2 (2.7)	5.4 (2.3)	< 0.001	< 0.001
If you contracted mpox, how much control would you feel you have over it (personal control) ^4^	6.7 (2.9)	6.2 (2.2)	< 0.001	0.001
If you contracted mpox, how much do you think available treatment could help (treatment control) ^5^	7.2 (2.7)	7.4 (1.9)	0.13	0.23
If you contracted mpox, how many symptoms would you expect to experience (identity) ^6^	7.6 (2.6)	7.5 (1.9)	0.60	0.23
If you contracted mpox, how concerned would you be (concern) ^7^	8.2 (2.6)	7.7 (2.4)	0.004	0.01
If you contracted mpox, how well do you feel you understand it (coherence) ^8^	5.5 (3.1)	4.9 (2.2)	< 0.001	< 0.001
If you contracted mpox, how much would it affect you emotionally (emotion) ^9^	7.8 (2.7)	7.8 (2.1)	0.82	0.33

^1^ Adjusted p values: p values obtained from multivariable logistic, linear or negative binomial regression after adjusting for background characteristics with significant between-group difference in Table 1 (age group, current relationship status, monthly personal income, sexual orientation, number of doses of COVID-19 vaccination received by the participants, mpox vaccination uptake, any type of HIV testing uptake, use of pre-exposure prophylaxis, testing for other sexually transmitted infections and use of other HIV-related services)

^2^ Consequences, item score: 0–10, a higher score indicated perceived more severe consequences of mpox infection

^3^ Timeline, item score: 0–10, a higher score indicated perceived mpox infection would last longer

^4^ Personal control, item score: 0–10, a higher score indicated the perception that one had stronger ability to control the mpox infection

^5^ Treatment control, item score: 0–10, a higher score indicated the perception that the treatment had stronger ability to control the mpox infection

^6^ Identity, item score: 0–10, a higher score indicated that one would have more symptoms of mpox infection

^7^ Concern, item score: 0–10, a higher score indicated that one had more concern about mpox infection

^8^ Coherence, item score: 0–10, a higher score indicated that one had more understanding about mpox

^9^ Emotion, item score: 0–10, a higher score indicated that one had more negative emotions caused by mpox infection

The mean (SD) of the items related to illness representations of mpox were presented in Table 2. Compared with GBMSM in Hong Kong, those in Beijing perceived more severe consequences (7.9 [2.7] vs. 7.3 [2.2]; *p* < 0.001), longer expected illness timeline (7.2 [2.7] vs. 5.4 [2.3]; *p* < 0.001), greater personal control (6.7 [2.9] vs. 6.2 [2.2]; *p* = 0.001), more concern (8.2 [2.6] vs. 7.7 [2.4]; *p* = 0.01), and better understanding related to mpox (coherence: 5.5 [3.1] vs. 4.9 [2.2]; *p* < 0.001). However, the scores of treatment control, identity, and emotional response were comparable between GBMSM in Beijing and Hong Kong.

### Factors associated with sexual behaviors

In univariate analyses, PrEP use was consistently associated with a higher prevalence of CAS with men and SDU, as well as a higher number of male sex partners in both Beijing and Hong Kong (Table 3). HIV testing uptake was associated with a higher prevalence of CAS with men in Hong Kong and a higher prevalence of SDU in Beijing, and was also associated with a higher number of male sex partners in both cities. Similarly, STIs testing was associated with a higher prevalence of CAS with men in Hong Kong and a higher prevalence of SDU, as well as a higher number of male sex partners, in both cities. Use of other HIV-related services was associated with a higher number of male sex partners in both cities. A history of confirmed SARS-CoV-2 infection was associated with a higher number of male sex partners in both cities and with a higher prevalence of CAS with men in Beijing. Participants with a higher monthly personal income had a greater number of male sex partners in both cities. Several city-specific associations were also observed. In Beijing, being married or cohabiting with either a male or female partner was associated with a higher prevalence of CAS with men and a higher number of male sex partners. Older age was also associated with having a greater number of male sex partners in Beijing. In Hong Kong, uptake of mpox vaccination was associated with a higher number of male sex partners among GBMSM.

**Table 3:** Associations between background characteristics and sexual behaviors

	CAS with men		Number of male sex partners with anal intercourse		Sexualized drug use	
	Beijing	Hong Kong	Beijing	Hong Kong	Beijing	Hong Kong
	OR (95% CI) p value	OR (95% CI) p value	IRR (95% CI) p value	IRR (95% CI, p value)	OR (95% CI, p value)	OR (95%CI, p value)
Age group (years)						
18–24	Reference	Reference	Reference	Reference	Reference	Reference
25–34	0.85 (0.49–1.45) *p* = 0.54	0.89 (0.52–1.51) *p* = 0.66	1.32 (0.97–1.80) *p* = 0.08	1.14 (0.85–1.53) *p* = 0.40	0.98 (0.49–1.97) *p* = 0.96	0.56 (0.24–1.28) *p* = 0.17
35–44	1.10 (0.61–1.97) *p* = 0.75	0.85 (0.49–1.50) *p* = 0.58	2.08 (1.49–2.90) *p* < 0.001	1.29 (0.94–1.76) *p* = 0.11	1.22 (0.58–2.57) *p* = 0.60	0.99 (0.43–2.27) *p* = 0.98
45 or above	1.58 (0.72–3.45) *p* = 0.26	0.69 (0.37–1.27) *p* = 0.23	2.21 (1.43–3.41) *P* < 0.001	1.15 (0.81–1.62) *p* = 0.43	0.79 (0.27–2.29) *p* = 0.66	0.53 (0.19–1.51) *p* = 0.23
Current relationship status						
Currently single	Reference	Reference	Reference	Reference	Reference	Reference
Married or cohabited with a man	3.08 (2.00–4.73) *p* < 0.001	1.54 (0.99–2.38) *p* = 0.054	1.36 (1.08–1.71) *p* = 0.003	0.94 (0.74–1.19) *p* = 0.61	1.16 (0.69–1.96) *p* = 0.57	1.19 (0.59–2.39) *p* = 0.62
Married or cohabited with a woman	2.32 (1.15–4.68) *p* = 0.02	0.21 (0.02–1.88) *p* = 0.16	1.36 (1.08–1.71) *p* = 0.008	0.42 (0.14–1.30) *p* = 0.13	0.95 (0.38–2.39) *p* = 0.92	2.54 (0.28–23.26) *p* = 0.41
Highest education level attained						
Senior high or below	Reference	Reference	Reference	Reference	Reference	Reference
College or above	0.90 (0.54–1.49) *p* = 0.68	1.24 (0.80–1.91) *p* = 0.33	1.06 (0.80–1.40) *p* = 0.71	1.10 (0.86–1.41) *p* = 0.43	0.86 (0.46–1.63) *p* = 0.65	1.20 (0.55–2.62) *p* = 0.65
Having a full-time employment						
Yes	Reference	Reference	Reference	Reference	Reference	Reference
No	0.78 (0.51–1.20) *p* = 0.26	1.17 (0.80–1.72) *p* = 0.41	0.74 (0.58–0.94) *p* = 0.013	1.02 (0.82–1.26) *p* = 0.88	0.56 (0.30–1.04) *p* = 0.07	1.51 (0.83–2.76) *p* = 0.18
Monthly personal income						
Below median income level	Reference	Reference	Reference	Reference	Reference	Reference
Median income or above	1.05 (0.73–1.52) *p* = 0.78	1.09 (0.77–1.54) *p* = 0.63	1.28 (1.05–1.57) *p* = 0.02	1.34 (1.10–1.63) *p* = 0.004	1.21 (0.75–1.95) *p* = 0.43	0.61 (0.35–1.06) *p* = 0.08
Refuse to disclose	0.76 (0.36–1.59) *p* = 0.46	0.83 (0.12–6.04) *p* = 0.86	0.68 (0.44–1.03) *p* = 0.07	1.74 (0.59–5.15) *p* = 0.31	1.27 (0.51–3.15) *p* = 0.61	2.38 (0.24–23.82) *p* = 0.46
Sexual orientation						
Gay	Reference	Reference	Reference	Reference	Reference	Reference
Bisexual	0.93 (0.58–1.51) *p* = 0.78	0.74 (0.43–1.27) *p* = 0.27	0.90 (0.69–1.18) *p* = 0.46	0.93 (0.69–1.27) *p* = 0.66	0.97 (0.52–1.79) *p* = 0.92	0.90 (0.35–2.36) *p* = 0.84
Heterosexual	N.A.	N.A.	0.07 (0.01–0.32) *p* < 0.001	0.93 (0.19–4.49) *p* = 0.94	N.A.	9.77 (0.60–158.46) *p* = 0.11
Uncertain	0.41 (0.18–0.93) *p* = 0.03	0.38 (0.03–4.25) *p* = 0.43	0.54 (0.35–0.83) *p* = 0.005	0.80 (0.22–2.96) *p* = 0.74	0.63 (0.22–1.86) *p* = 0.40	N.A.
Self-reported to be HIV positive						
No	Reference	Reference	Reference	Reference	Reference	Reference
Yes	1.13 (0.55–2.31) *p* = 0.74	1.32 (0.31–5.56) *p* = 0.71	1.02 (0.69–1.52) *p* = 0.92	1.31 (0.61–2.82) *p* = 0.49	1.27 (0.53–3.02) *p* = 0.59	1.40 (0.17–11.59) *p* = 0.76
History of confirmed SARS-CoV-2 infection						
No	Reference	Reference	Reference	Reference	Reference	Reference
Yes	1.90 (1.16–3.14) *p* = 0.01	1.43 (0.95–2.17) *p* = 0.09	1.32 (1.01–1.73) *p* = 0.04	1.29 (1.02–1.64) *p* = 0.04	1.56 (0.79–3.08) *p* = 0.20	1.36 (0.63–2.96) *p* = 0.44
Number of doses of COVID-19 vaccination received by the participants						
0	Reference	Reference	Reference	Reference	Reference	Reference
1	1.05 (0.20–5.60) *p* = 0.95	0.91 (0.18–4.50) *p* = 0.91	0.76 (0.31–1.86) *p* = 0.55	1.22 (0.50–3.01) *p* = 0.66	1.50 (0.22–10.36) *p* = 0.68	0.67 (0.06–7.48) *p* = 0.74
2	1.64 (0.62–4.29) *p* = 0.32	1.88 (0.67–5.24) *p* = 0.23	0.92 (0.55–1.53) *p* = 0.75	1.38 (0.78–2.45) *p* = 0.26	1.48 (0.45–4.84) *p* = 0.52	0.47 (0.11–2.03) *p* = 0.31
3	1.35 (0.55–3.28) *p* = 0.51	0.80 (0.32–2.04) *p* = 0.65	0.68 (0.43–1.09) *p* = 0.11	1.22 (0.72–2.08) *p* = 0.45	0.89 (0.29–2.72) *p* = 0.84	0.55 (0.15–1.96) *p* = 0.36
4	1.60 (0.49–5.29) *p* = 0.44	0.95 (0.34–2.62) *p* = 0.92	1.84 (0.98–3.44) *p* = 0.06	1.28 (0.72–2.27) *p* = 0.41	0.95 (0.21–4.37) *p* = 0.95	0.54 (0.13–2.33) *p* = 0.41
5	1.31 (0.23–7.41) *p* = 0.76	0.73 (0.08–6.31) *p* = 0.73	0.54 (0.21–1.44) *p* = 0.22	0.83 (0.23–2.97) *p* = 0.78	1.80 (0.25–12.85) *p* = 0.56	N.A.
Uptake of mpox vaccination						
No	Reference	Reference	Reference	Reference	Reference	Reference
Yes	N.A.	1.25 (0.85–1.83) *p* = 0.25	0.58 (0.20–1.65) *p* = 0.58	2.35 (1.91–2.88) *p* < 0.001	1.12 (0.12–10.10) *p* = 0.92	1.45 (0.80–2.65) *p* = 0.23
Use of any types of HIV testing						
No	Reference	Reference	Reference	Reference	Reference	Reference
Yes	1.22 (0.75–1.98) *p* = 0.43	1.81 (1.31–2.51) *p* < 0.001	1.71 (1.30–2.26) *p* < 0.001	1.79 (1.50–2.14) *p* < 0.001	3.87 (1.52–9.84) *p* = 0.01	1.67 (0.96–2.90) *p* = 0.07
Use of pre-exposure prophylaxis						
No	Reference	Reference	Reference	Reference	Reference	Reference
Yes	2.80 (1.78–4.39) *p* < 0.001	7.65 (3.88–15.11) *p* < 0.001	1.98 (1.56–2.50) *p* < 0.001	2.39 (1.88–3.05) *p* < 0.001	2.60 (1.58–4.27) *p* < 0.001	7.98 (4.45–14.32) *p* < 0.001
Testing for other sexually transmitted infections						
No	Reference	Reference	Reference	Reference	Reference	Reference
Yes	1.30 (0.91–1.86) *p* = 0.16	1.80 (1.29–2.52) *p* < 0.001	2.00 (1.63–2.45) *p* < 0.001	2.06 (1.72–2.48) *p* < 0.001	2.17 (1.30–3.63) *p* = 0.003	2.55 (1.46–4.45) *p* < 0.001
Use of other HIV-related services						
No	Reference	Reference	Reference	Reference	Reference	Reference
Yes	1.16 (0.82–1.65) *p* = 0.41	1.25 (0.91–1.72) *p* = 0.18	1.90 (1.56–2.32) *p* = 0.001	1.43 (1.20–1.71) *p* < 0.001	1.27 (0.80–2.02) *p* = 0.30	1.67 (0.97–2.90) *p* = 0.07

OR: odds ratio

IRR: incidence rate ratios

After adjusting for the significant background characteristics, better understanding of mpox (coherence) was associated with a lower likelihood of CAS with men (AOR = 0.92, 95% CI: 0.87–0.98, *p* = 0.01) among GBMSM in Beijing (Table 4). In addition, longer expected illness timeline (timeline) (IRR = 0.96, 95% CI: 0.93–1.00, *p* = 0.049), anticipating more severe symptoms if infected (identity) (IRR = 0.94, 95% CI: 0.90–0.97, *p* < 0.001) and stronger negative emotions (emotion) (IRR = 0.94, 95% CI: 0.90–0.98, *p* = 0.01) were associated with fewer male sex partners among GBMSM in the same city. In contrast, greater personal control (IRR = 1.06, 95% CI: 1.02–1.10, *p* = 0.001) was associated with higher number of male sex partners in Beijing. However, the associations between all domains of mpox illness representations and the studied sexual behaviors were not of statistically significant among GBMSM in Hong Kong. All multivariable models showed no evidence of multicollinearity (all VIF < 5). Similarly, no lack of fit was detected among all multivariable models.

**Table 4:** Associations between mpox disease perceptions and sexual behaviors

	CAS with men		Number of male sex partners with anal intercourse		Sexualized drug use	
	Beijing	Hong Kong	Beijing	Hong Kong	Beijing	Hong Kong
	AOR ^1^ (95% CI, p value)	AOR ^2^ (95% CI, p value)	Adjusted IRR ^3^ (95% CI, p value)	Adjusted IRR ^4^ (95% CI, p value)	AOR ^5^ (95% CI, p value)	AOR ^6^ (95% CI, p value)
Domains of the Brief Illness Perception Questionnaire (B-IPQ), mean (SD)						
Consequences	1.02 (0.95–1.09) *p* = 0.63	0.94 (0.87–1.02) *p* = 0.13	0.98 (0.94–1.02) *p* = 0.27	1.04 (0.99–1.09) *p* = 0.054	0.94 (0.87–1.02) *p* = 0.12	0.95 (0.84–1.08) *p* = 0.44
Timeline	1.05 (0.97–1.11) *p* = 0.32	0.97 (0.90–1.04) *p* = 0.39	**0.96 (0.93**–**1.00)** *p* = 0.049	1.00 (0.97–1.05) *p* = 0.69	0.97 (0.89–1.05) *p* = 0.42	1.01 (0.89–1.14) *p* = 0.86
Personal control	1.01 (0.94–1.08) *p* = 0.82	1.01 (0.94–1.09) *p* = 0.82	**1.06 (1.02**–**1.10)** *p* = 0.001	1.04 (1.00–1.09) *p* = 0.052	1.04 (0.95–1.12) *p* = 0.41	0.94 (0.82–1.07) *p* = 0.32
Treatment control	1.05 (0.98–1.12) *p* = 0.18	1.01 (0.92–1.10) *p* = 0.89	0.99 (0.96–1.03) *p* = 0.57	0.97 (0.93–1.02) *p* = 0.23	0.99 (0.91–1.07) *p* = 0.74	0.94 (0.81–1.08) *p* = 0.37
Identity	1.01 (0.94–1.09) *p* = 0.79	0.94 (0.86–1.02) *p* = 0.14	**0.94 (0.90**–**0.97)** *p* < 0.001	1.04 (0.99–1.09) *p* = 0.09	0.95 (0.87–1.03) *p* = 0.22	0.91 (0.79–1.05) *p* = 0.91
Concern	1.05 (0.98–1.13) *p* = 0.18	0.97 (0.91–1.04) *p* = 0.44	0.97 (0.93–1.00) *p* = 0.08	0.96 (0.93–1.00) *p* = 0.07	0.94 (0.86–1.02) *p* = 0.12	0.99 (0.87–1.13) *p* = 0.92
Coherence	**0.92 (0.87**–**0.98)** *p* = 0.01	0.95 (0.88–1.02) *p* = 0.16	1.02 (0.99–1.05) *p* = 0.31	1.00 (0.96–1.04) *p* = 0.95	0.96 (0.89–1.03) *p* = 0.23	0.92 (0.80–1.05) *p* = 0.21
Emotion	1.04 (0.97–1.12) *p* = 0.23	0.99 (0.92–1.08) *p* = 0.85	**0.94 (0.90**–**0.98)** *p* = 0.001	1.02 (0.97–1.06) *p* = 0.46	0.93 (0.86–1.01) *p* = 0.08	0.93 (0.82–1.05) *p* = 0.25

^1^ AOR: adjusted odds ratios, odds ratios adjusted for background characteristics that were significantly associated with CAS with men among participants in Beijing, including current relationship status, sexual orientation, history of confirmed SARS-CoV-2 infection, use of pre-exposure prophylaxis

^2^ AOR: adjusted odds ratios, odds ratios adjusted for background characteristics that were significantly associated with CAS with men among participants in Hong Kong, including use of any types of HIV testing, use of pre-exposure prophylaxis, and testing for other sexually transmitted infections

^3^ Adjusted IRR: incidence rate ratio adjusted for background characteristics that were significantly associated with number of male sex partners with anal intercourse among MSM in Beijing, including age group, relationship status, employment status, personal income, sexual orientation, history of SARS-CoV-2 infection, use of any types of HIV testing, use of pre-exposure prophylaxis, testing for other sexually transmitted infections, and use of other HIV-related services

^4^ Adjusted IRR: incidence rate ratio adjusted for background characteristics that were significantly associated with number of male sex partners with anal intercourse among MSM in Hong Kong, including monthly personal income, history of SARS-CoV-2 infection, uptake of mpox vaccination, use of any types of HIV testing, use of pre-exposure prophylaxis, testing for other sexually transmitted infections, and use of other HIV-related services

^5^ AOR: adjusted odds ratios, odds ratios adjusted for background characteristics that were significantly associated with sexualized drug use among participants in Beijing, including use of any types of HIV testing, use of pre-exposure prophylaxis, and testing for other sexually transmitted infections

^6^ AOR: adjusted odds ratios, odds ratios adjusted for background characteristics that were significantly associated with sexualized drug use among participants in Hong Kong, including use of pre-exposure prophylaxis and testing for other sexually transmitted infections

### Sensitivity analysis of the associations between mpox illness representation and sexual behaviors among GBMSM who have never received mpox vaccination

Unvaccinated GBMSM in Beijing perceived more severe consequences (7.9 [2.7] vs. 7.3 [2.2]; *p* < 0.001), longer expected illness timeline (7.2 [2.7] vs. 5.5 [2.2]; *p* < 0.001), greater personal control (6.7 [2.9] vs. 6.1 [2.2]; *p* = 0.001), more concern (8.2 [2.6] vs. 7.6 [2.4]; *p* < 0.001), and better understanding related to mpox (coherence: 5.5 [3.1] vs. 4.5 [2.1]; *p* < 0.001) comparing to unvaccinated GBMSM in Hong Kong. The associations between BIPQ domains and the dependent variables among unvaccinated participants were similar to those obtained from all GBMSM in both cities, with the exception of consequence and identity. Perceived more severe consequences (consequences) (IRR = 1.07, 95% CI: 1.01–1.12, *p* = 0.01) and anticipating more severe symptoms if infected (identity) (IRR = 1.09, 95% CI: 1.03–1.15, *p* = 0.002) were significantly associated with number of sex partners among unvaccinated participants in Hong Kong, but not among all Hong Kong GBMSM (Supplementary Material 1).

## Discussion

This is one of the first studies exploring how mpox illness representations would affect sexual behaviors among GBMSM. Existing studies on illness representations have focused on vaccination, screening, or self-care in other health conditions. Our findings expanded the application of illness representation in explaining health-related behaviors. The results supported our hypotheses that GBMSM in Beijing and Hong Kong had different levels of illness perceptions related to mpox. Associations between mpox-related illness perceptions and sexual behaviors were observed between GBMSM in Beijing, but were not detected in Hong Kong. Comparing two study sites with different mpox situation, a relatively large sample size and high response rate were other strengths of this study.

About half of the participants in both cities reported CAS and 10–20% reported SDU in the past six months. As compared to studies conducted in 2018–2019, increasing trends in CAS with men and SDU were observed among GBMSM in both cities [18, 41]. Given the limited coverage of PrEP in both cities (18.3% in Beijing and 14.3% in Hong Kong), our participants were still at a relatively high risk of HIV transmission. These sexual behaviors also contributed to the increasing burdens caused by other STIs in both cities [41, 42]. Therefore, effective and innovative strategies are needed to reduce CAS with men, number of male sex partners or SDU among GBMSM in both Beijing and Hong Kong.

In line with our hypotheses, the levels of illness perceptions related to mpox were different between GBMSM in Beijing and Hong Kong. As compared to GBMSM in Hong Kong, those in Beijing perceived mpox would have stronger negative influences on their lives (consequences) and long duration of infection (timeline). The mpox vaccine is effective in preventing mpox infection and protecting against severe symptoms [43]. Mpox vaccination is available in Hong Kong and about one quarter of the GBMSM in this city already received such vaccination. In contrast, such vaccination is not available in Beijing and the vaccination uptake rate was minimal. Therefore, more GBMSM in Hong Kong might feel that they are protected by the vaccines than their counterparts in Beijing. Even infected with mpox, the impacts and duration of the infection would be milder under the protection conferred by the vaccines. In addition to the unavailability of the vaccine, mainland China including Beijing has more mpox cases than that of Hong Kong. It is expected that GBMSM in Beijing would be more concerned about mpox than their counterparts in Hong Kong, and hence have more understanding about the infection. Without effective vaccines, GBMSM in Beijing can only rely on themselves to prevent mpox. This could partially explain why the levels of personal control was higher among GBMSM in Beijing comparing to their counterparts in Hong Kong.

Associations between mpox-related illness perceptions and sexual behaviors were observed between GBMSM in Beijing, but were not detected in Hong Kong. As reflected by the higher levels of illness representation, it is possible that GBMSM in Beijing would consider mpox as a serious health threat. They might consider changing their sexual behaviors as coping strategies to regulate illness representation. However, for GBMSM in Hong Kong, their levels of illness representation were lower and might not reach the threshold to trigger coping strategies (e.g., change sexual behaviors). Our findings provided some insight to develop health communication messages to reduce CAS with men and number of male sex partners among GBMSM in Beijing. Those who had a more thorough understanding (coherence) of mpox were less likely to report CAS with men. Similar association between coherence of HIV and sexual behaviors were observed in a previous study [44]. Longer expected illness timeline (timeline) and perceived severe symptoms following mpox infection (identity) to mpox were associated with fewer number of male sex partners in Beijing. Similar associations between illness timeline perceptions and sexual behaviors have been reported in the context of HIV [45]. Studies in the US and Europe showed that perceived severity of mpox was associated with reductions in sexual behaviors among GBMSM [11, 46]. These results provided some implications for generating hypotheses. Future studies may test whether delivering accurate mpox-related information (e.g., transmission of mpox through intimate and sexual contacts, expected timeline of mpox infection, and severe symptoms following mpox infection) are helpful to reduce CAS and/or number of sex partners among GBMSM in Beijing. In addition, mpox infection clearly brings about negative emotions in many GBMSM. Higher levels of these negative emotions were correlated with lower number of male sex partners. Future programs may consider framing the reduction of number of sex partners as a positive coping strategy in order to lower the levels of negative emotions among GBMSM. In contrast, greater perceived personal control over mpox was associated with a higher number of male sex partners in Beijing. This finding may reflect greater prevention self-efficacy or confidence in personal control, which could be associated with more male sex partners.

In the sensitivity analysis, perceived more severe consequences and anticipating more severe symptoms if infected were significantly associated with number of sex partners among unvaccinated participants in Hong Kong, but not in the entire sample of GBMSM in the same city. Previous studies showed that uptake of mpox vaccination was associated with mpox-related illness representation, information exposure, risk perceptions, and other HIV-related service utilization among GBMSM in Hong Kong [5, 27]. These factors could also influence the onset of sexual behaviors among GBMSM in the same city.

This study has several limitations. First, participants were recruited through convenience sampling. The samples might not represent GBMSM in Beijing or Hong Kong. Moreover, due to differences in healthcare system, health literacy and cultural factors, the findings may not be generalizable to GBMSM populations in other Chinese cities or international contexts. Second, the measurement tool was adapted from the validated Chinese version of BIPQ and its internal consistency was acceptable. However, we did not validate the adapted tool in a separated study. Third, data could not be collected from individuals who declined participation. Their characteristics may differ from those who participated, introducing potential selection bias. However, the relatively high response rate may reduce this concern. Fourth, social desirability bias might exist. Participant might under-report CAS with men, number of male sex partners or SDU and over-report preventive behaviors. Fifth, this study did not include social media influencing factors in this study. It is possible that social media use would affect sexual behaviors through mpox illness perceptions. In addition, the cross-sectional design could not establish causal inference, and future longitudinal studies are needed to clarify the temporal relationships between mpox illness representations and sexual behaviors.

## Conclusion

GBMSM in Beijing had higher levels of illness representation related to mpox comparing to their counterparts in Hong Kong. Illness representations were associated with sexual behaviors among GBMSM in Beijing and the associations were not detected in Hong Kong. The findings provided implications for generating hypotheses for future interventions. Future studies may explore whether modifying mpox-related illness representation is helpful to reduce CAS and number of male sex partners among GBMSM in Beijing, such as increasing coherence, disseminating information related to timeline of infection and severe symptoms, and guiding those with more negative emotions to adopt safe sex practice as positive coping strategies.

## Supplementary Information

Below is the link to the electronic supplementary material.

Supplementary Material 1: Sensitivity analysis of the associations between mpox illness representation and sexual behaviors among GBMSM who have never received mpox vaccination

## Data Availability

The datasets generated or analyzed during this study are not publicly available because they contain sensitive personal behaviors but are available from the corresponding author on reasonable request.

## Abbreviations

GBMSM: Gay bisexual and other men who have sex with men
CAS: Condomless anal sex
SDU: Sexualized drug use
STIs: Sexually transmitted infections
PrEP: Pre-exposure prophylaxis
BIPQ: Brief Illness Perception Questionnaire
AORs: Adjusted odds ratios
β: Standardized coefficients
VIF: Variance inflation factor
CI: Confidence interval
SD: Standard deviation
