# Supplementary material for: Differential influences of mpox illness representation on sexual behaviors among gay, bisexual, and other men who have sex with men in Beijing and Hong Kong
Source: BMC Infect Dis. 2026 Mar 13;26:801. doi: 10.1186/s12879-026-13062-7 (PMC13101376; doi:10.1186/s12879-026-13062-7)
Supplement: Supplementary file 1 — Supplementary Material 1: Sensitivity analysis of the associations between mpox illness representation and sexual behaviors among GBMSM who have never received mpox vaccination [file 12879_2026_13062_MOESM1_ESM.docx]

Additional file 1. Sensitivity analysis of the associations between mpox illness representation and sexual behaviors among GBMSM who have never received mpox vaccination

Table S1. Background characteristics of GBMSM without a history of mpox vaccination in Beijing and Hong Kong, China

|  | Beijing  (n=519) | Hong Kong (n=470) | p value |
| --- | --- | --- | --- |
|  | n (%) | n (%) |  |
| **Socio-demographic characteristics** |  |  |  |
| Age group (years) |  |  |  |
| 18-24 | 64 (12.3) | 66 (14.1) |  |
| 25-34 | 276 (53.2) | 212 (45.1) |  |
| 35-44 | 138 (26.6) | 118 (25.1) |  |
| 45 or above | 41 (7.9) | 74 (15.7) | <0.001 |
| Current relationship status |  |  |  |
| Currently single | 365 (70.4) | 387 (82.4) |  |
| Married or cohabited with a man | 119 (22.9) | 80 (17.0) |  |
| Married or cohabited with a woman | 35 (6.7) | 3 (0.6) | <0.001 |
| Highest education level attained |  |  |  |
| Senior high or below | 68 (13.1) | 85 (18.1) |  |
| College or above | 451 (86.9) | 385 (81.9) | 0.03 |
| Having a full-time employment |  |  |  |
| Yes | 414 (79.8) | 360 (76.6) |  |
| No | 105 (20.2) | 110 (23.4) | 0.23 |
| Monthly personal income |  |  |  |
| Below median income level ^1^ | 190 (36.6) | 149 (31.7) |  |
| Median income or above | 294 (56.7) | 318 (67.7) |  |
| Refuse to disclose | 35 (6.7) | 3 (0.6) | <0.001 |
| Sexual orientation |  |  |  |
| Gay | 403 (77.6) | 420 (89.4) |  |
| Bisexual | 81 (15.6) | 46 (9.8) |  |
| Heterosexual | 6 (1.2) | 1 (0.2) |  |
| Uncertain | 29 (5.6) | 3 (0.6) | <0.001 |
| Self-reported to be HIV positive |  |  |  |
| No | 488 (94.0) | 470 (100.0) |  |
| Yes | 31 (6.0) | 0 (0.0) | <0.001 |
| History of confirmed SARS-CoV-2 infection |  |  |  |
| No | 81 (15.6) | 90 (19.1) |  |
| Yes | 438 (84.4) | 380 (80.9) | 0.14 |
| Number of doses of COVID-19 vaccination received by the participants |  |  |  |
| 0 | 22 (4.2) | 17 (3.6) |  |
| 1 | 8 (1.5) | 7 (1.5) |  |
| 2 | 89 (17.1) | 68 (14.5) |  |
| 3 | 371 (71.6) | 328 (69.8) |  |
| 4 | 23 (4.4) | 47 (10.0) |  |
| 5 | 6 (1.2) | 3 (0.6) | 0.02 |
| **HIV-related services utilization in the past six months** |  |  |  |
| Use of any types of HIV testing |  |  |  |
| No | 79 (15.2) | 285 (60.6) |  |
| Yes | 440 (84.8) | 185 (39.4) | <0.001 |
| Use of pre-exposure prophylaxis |  |  |  |
| No | 419 (80.7) | 431 (91.7) |  |
| Yes | 100 (19.3) | 39 (8.3) | <0.001 |
| Testing for other sexually transmitted infections |  |  |  |
| No | 187 (36.0) | 322 (68.5) |  |
| Yes | 332 (64.0) | 148 (31.5) | <0.001 |
| Use of other HIV-related services |  |  |  |
| No | 207 (39.9) | 275 (58.5) |  |
| Yes | 312 (60.1) | 195 (41.5) | <0.001 |

^1^ Median income of the residents was ¥7,000 (US$980) per month in Beijing and HK$20,000 (US$2,564) per month in Hong Kong

Table S2 Sexual behaviors and mpox disease perceptions

|  | Beijing  (n=519) | Hong Kong  (n=470) | Unadjusted  p value | Adjusted p value ^1^ |
| --- | --- | --- | --- | --- |
|  | n (%) | n (%) |  |  |
| **Sexual behaviors in the past six months** |  |  |  |  |
| Condomless anal sex with men |  |  |  |  |
| No | 288 (55.5) | 213 (45.3) |  |  |
| Yes | 231 (44.5) | 257 (54.7) | 0.001 | <0.001 |
| Number of male sex partners with anal intercourse |  |  |  |  |
| Mean (SD) | 4.2 (8.4) | 2.8 (4.0) | 0.002 | 0.64 |
| Sexualized drug use |  |  |  |  |
| No | 424 (81.7) | 430 (91.5) |  |  |
| Yes | 95 (18.3) | 40 (8.5) | <0.001 | 0.15 |
| **Mpox disease perceptions** |  |  |  |  |
| Domains of the Brief Illness Perception Questionnaire (B-IPQ), mean (SD) |  |  |  |  |
| If you contracted mpox, how much would it affect your life (consequences) ^2^ | 7.9 (2.7) | 7.3 (2.2) | <0.001 | <0.001 |
| If you contracted mpox, how long do you think it would last (timeline) ^3^ | 7.2 (2.7) | 5.5 (2.2) | <0.001 | <0.001 |
| If you contracted mpox, how much control would you feel you have over it (personal control) ^4^ | 6.7 (2.9) | 6.1 (2.1) | <0.001 | 0.004 |
| If you contracted mpox, how much do you think available treatment could help (treatment control) ^5^ | 7.2 (2.7) | 7.4 (1.8) | 0.17 | 0.16 |
| If you contracted mpox, how many symptoms would you expect to experience (identity) ^6^ | 7.5 (2.6) | 7.4 (1.9) | 0.28 | 0.27 |
| If you contracted mpox, how concerned would you be (concern) ^7^ | 8.2 (2.6) | 7.6 (2.5) | <0.001 | 0.007 |
| If you contracted mpox, how well do you feel you understand it (coherence) ^8^ | 5.5 (3.1) | 4.5 (2.1) | <0.001 | <0.001 |
| If you contracted mpox, how much would it affect you emotionally (emotion) ^9^ | 7.8 (2.7) | 7.7 (2.1) | 0.49 | 0.30 |

^1^ Adjusted p values: p values obtained from multivariable logistic, linear or negative binomial regression after adjusting for background characteristics with significant between-group difference in Table 1 (age group, current relationship status, highest education level, monthly personal income, sexual orientation, self-reported to be HIV positive, number of doses of COVID-19 vaccination received by the participants, any type of HIV testing uptake, use of pre-exposure prophylaxis, testing for other sexually transmitted infections and use of other HIV-related services)

^2^ Consequences, item score: 0-10, a higher score indicated perceived more severe consequences of mpox infection

^3^ Timeline, item score: 0-10, a higher score indicated perceived mpox infection would last longer

^4^ Personal control, item score: 0-10, a higher score indicated the perception that one had stronger ability to control the mpox infection

^5^ Treatment control, item score: 0-10, a higher score indicated the perception that the treatment had stronger ability to control the mpox infection

^6^ Identity, item score: 0-10, a higher score indicated that one would have more symptoms of mpox infection

^7^ Concern, item score: 0-10, a higher score indicated that one had more concern about mpox infection

^8^ Coherence, item score: 0-10, a higher score indicated that one had more understanding about mpox

^9^ Emotion, item score: 0-10, a higher score indicated that one had more negative emotions caused by mpox infection

Table S3 Associations between background characteristics and sexual behaviors

|  | CAS with men | | Number of male sex partners with anal intercourse | | Sexualized drug use | |
| --- | --- | --- | --- | --- | --- | --- |
|  | Beijing | Hong Kong | Beijing | Hong Kong | Beijing | Hong Kong |
|  | OR (95% CI)  p value | OR (95% CI)  p value | IRR (95% CI)  p value | IRR (95% CI,  p value) | OR (95% CI,  p value) | OR (95% CI,  p value) |
| Age group (years) |  |  |  |  |  |  |
| 18-24 | Reference | Reference | Reference | Reference | Reference | Reference |
| 25-34 | 0.79 (0.46–1.36)  p=0.39 | 0.72 (0.41–1.27)  p=0.25 | 1.35 (0.98–1.85)  p=0.06 | 0.78 (0.57–1.08)  p=0.14 | 0.91 (0.45–1.84)  p=0.80 | 0.45 (0.18–1.09)  p=0.08 |
| 35-44 | 1.01 (0.56–1.83)  p=0.98 | 0.61 (0.33–1.13)  p=0.12 | 2.12 (1.51–2.97)  p<0.001 | 0.86 (0.61–1.21)  p=0.38 | 1.15 (0.55–2.54)  p=0.71 | 0.65 (0.26–1.66)  p=0.37 |
| 45 or above | 1.45 (0.66–3.19)  p=0.36 | 0.54 (0.28–1.07)  p=0.08 | 2.25 (1.45–3.49)  p<0.001 | 1.00 (0.69–1.47)  p=0.99 | 0.74 (0.26–2.17)  p=0.59 | 0.56 (0.19–1.66)  p=0.30 |
| Current relationship status |  |  |  |  |  |  |
| Currently single | Reference | Reference | Reference | Reference | Reference | Reference |
| Married or cohabited with a man | 3.01(1.96–4.63)  p<0.001 | 1.76 (1.06–2.92)  p=0.03 | 1.35 (1.08–1.70)  p=0.009 | 0.82 (0.62–1.09)  p=0.06 | 1.17 (0.69–1.97)  p=0.56 | 1.46 (0.66–3.19)  p=0.35 |
| Married or cohabited with a woman | 2.27 (1.13–4.59)  p=0.02 | N.A. | 1.75 (1.20–2.54)  p=0.004 | 0.11 (0.01–1.09)  p=0.06 | 0.96 (0.38–2.39)  p=0.92 | N.A. |
| Highest education level attained |  |  |  |  |  |  |
| Senior high or below | Reference | Reference | Reference | Reference | Reference | Reference |
| College or above | 0.89 (0.53–1.48)  p=0.65 | 1.15 (0.72–1.85)  p=0.55 | 1.04 (0.79–1.39)  p=0.71 | 0.96 (0.73–1.26)  p=0.75 | 0.84 (0.45–1.59)  p=0.60 | 0.87 (0.39–1.97)  p=0.87 |
| Having a full-time employment |  |  |  |  |  |  |
| Yes | Reference | Reference | Reference | Reference | Reference | Reference |
| No | 0.79 (0.51–1.23)  p=0.30 | 1.26 (0.82–1.95)  p=0.29 | 0.73 (0.57–0.93)  p=0.01 | 1.23 (0.96–1.57)  p=0.10 | 0.52 (0.27–0.98)  p=0.04 | 1.87 (0.94–3.73)  p=0.07 |
| Monthly personal income |  |  |  |  |  |  |
| Below median income level | Reference | Reference | Reference | Reference | Reference | Reference |
| Median income or above | 1.05 (0.71–1.47)  p=0.91 | 1.07 (0.72–1.58)  p=0.74 | 1.28 (1.04–1.57)  p=0.02 | 1.09 (0.87–1.37)  p=0.46 | 1.23 (0.76–2.00)  p=0.39 | 0.52 (0.27–1.00)  p=0.049 |
| Refuse to disclose | 0.73 (0.35–1.53)  p=0.41 | 0.43 (0.04–4.86)  p=0.50 | 0.67 (0.44–1.03)  p=0.07 | 2.52 (0.74–8.59)  p=0.14 | 1.28 (0.51–3.20)  p=0.59 | 3.64 (0.31–42.19)  p=0.30 |
| Sexual orientation |  |  |  |  |  |  |
| Gay | Reference | Reference | Reference | Reference | Reference | Reference |
| Bisexual | 0.92 (0.57–1.49)  p=0.75 | 0.74 (0.40–1.35)  p=0.32 | 0.90 (0.69–1.18)  p=0.45 | 0.84 (0.59–1.21)  p=0.66 | 0.98 (0.53–1.81)  p=0.94 | 0.47 (0.11–2.02)  p=0.31 |
| Heterosexual | N.A. | N.A. | 0.08 (0.02–0.38)  P=0.002 | 0.69 (0.06–7.67)  p=0.77 | N.A. | N.A. |
| Uncertain | 0.44 (0.19–1.02)  p=0.06 | 0.38 (0.03–4.25)  p=0.43 | 0.54 (0.35–0.85)  p=0.008 | 1.04 (0.28–3.86)  p=0.95 | 0.69 (0.23–2.04)  p=0.50 | N.A. |
| Self-reported to be HIV positive |  |  |  |  |  |  |
| No | Reference | Reference | Reference | Reference | Reference | Reference |
| Yes | 1.18 (0.57–2.44)  p=0.65 | N.A. | 1.04 (0.70–1.56)  p=0.83 | N.A. | 1.08 (0.43–2.70)  p=0.88 | N.A. |
| History of confirmed SARS-CoV-2 infection |  |  |  |  |  |  |
| No | Reference | Reference | Reference | Reference | Reference | Reference |
| Yes | 1.86 (1.13–3.08)  p=0.02 | 1.33 (0.84–2.11)  p=0.22 | 1.29 (0.99–1.69)  p=0.06 | 1.23 (0.94–1.62)  p=0.13 | 1.51 (0.77–2.98)  p=0.23 | 1.13 (0.48–2.64)  p=0.78 |
| Number of doses of COVID-19 vaccination received by the participants |  |  |  |  |  |  |
| 0 | Reference | Reference | Reference | Reference | Reference | Reference |
| 1 | 1.05 (0.20–5.60)  p=0.95 | 0.93 (0.16–5.54)  p=0.94 | 0.76 (0.31–1.86)  p=0.55 | 1.24 (0.46–3.36)  p=0.68 | 1.50 (0.22–10.36)  p=0.68 | N.A. |
| 2 | 1.64 (0.62–4.29)  p=0.32 | 1.94 (0.64–5.88)  p=0.24 | 0.92 (0.55–1.53)  p=0.75 | 1.18 (0.64–2.17)  p=0.60 | 1.48 (0.45–4.84)  p=0.52 | 0.45 (0.10–2.03)  p=0.30 |
| 3 | 1.38 (0.56–3.35)  p=0.49 | 0.72 (0.27–1.93)  p=0.51 | 0.68 (0.43–1.09)  p=0.11 | 0.93 (0.53–1.63)  p=0.80 | 0.89 (0.29–2.71)  p=0.83 | 0.42 (0.11–1.55)  p=0.19 |
| 4 | 1.60 (0.49–5.29)  p=0.44 | 0.80 (0.26–2.46)  p=0.69 | 1.84 (0.98–3.44)  p=0.06 | 0.71 (0.37–1.36)  p=0.30 | 0.95 (0.21–4.37)  p=0.95 | 0.43 (0.09–2.18)  p=0.31 |
| 5 | 1.75 (0.28–10.81)  p=0.55 | 1.40 (0.11–18.62)  p=0.80 | 0.41 (0.14–1.20)  p=0.10 | 0.22 (0.03–1.44)  p=0.12 | 2.25 (0.30–16.85)  p=0.43 | N.A. |
| Use of any types of HIV testing |  |  |  |  |  |  |
| No | Reference | Reference | Reference | Reference | Reference | Reference |
| Yes | 1.21 (0.75–1.97)  p=0.44 | 1.85 (1.27–2.70)  p=0.001 | 1.70 (1.28–2.24)  p<0.001 | 1.62 (1.31–2.01)  p<0.001 | 3.81 (1.49–9.69)  p=0.005 | 2.00 (1.04–3.85)  p=0.04 |
| Use of pre-exposure prophylaxis |  |  |  |  |  |  |
| No | Reference | Reference | Reference | Reference | Reference | Reference |
| Yes | 2.83 (1.80–4.46)  p<0.001 | 8.24 (2.88–23.58)  p<0.001 | 1.97 (1.55–2.49)  p<0.001 | 1.98 (1.38–2.84)  p<0.001 | 2.66 (1.62–4.38)  p<0.001 | 10.15 (4.74–21.73)  p<0.001 |
| Testing for other sexually transmitted infections |  |  |  |  |  |  |
| No | Reference | Reference | Reference | Reference | Reference | Reference |
| Yes | 1.28 (0.89–1.84)  p=0.18 | 2.01 (1.34–3.01)  p<0.001 | 1.99 (1.62–2.44)  p<0.001 | 1.81 (1.45–2.26)  p<0.001 | 2.11 (1.26–3.54)  p=0.004 | 2.95 (1.53–5.69)  p=0.001 |
| Use of other HIV-related services |  |  |  |  |  |  |
| No | Reference | Reference | Reference | Reference | Reference | Reference |
| Yes | 1.14 (0.80–1.63)  p=0.46 | 1.80 (1.24–2.62)  p=0.002 | 1.90 (1.55–2.32)  p<0.001 | 1.42 (1.15–1.76)  p=0.001 | 1.31 (0.82–2.08)  p=0.26 | 2.55 (1.31–4.98)  p=0.006 |

OR: odds ratio

IRR: incidence rate ratios

Table S4 Associations between mpox disease perceptions and sexual behaviors

|  | CAS with men | | Number of male sex partners with anal intercourse | | Sexualized drug use | |
| --- | --- | --- | --- | --- | --- | --- |
|  | Beijing | Hong Kong | Beijing | Hong Kong | Beijing | Hong Kong |
|  | AOR ^1^ (95% CI,  p value) | AOR ^2^ (95% CI,  p value) | Adjusted IRR ^3^ (95% CI,  p value) | Adjusted IRR ^4^ (95% CI,  p value) | AOR ^5^ (95% CI,  p value) | AOR ^6^ (95% CI,  p value) |
| Domains of the Brief Illness Perception Questionnaire (B-IPQ), mean (SD) |  |  |  |  |  |  |
| Consequences | 1.01 (0.94–1.08)  p=0.86 | 0.97 (0.89–1.06)  p=0.47 | 0.98 (0.94–1.02)  p=0.29 | **1.07 (1.01**–**1.12)**  **p=0.01** | 0.94 (0.86–1.02)  p=0.12 | 0.97 (0.83–1.13)  p=0.68 |
| Timeline | 1.02 (0.96–1.10)  p=0.49 | 1.00 (0.91–1.09)  p=0.93 | **0.96 (0.92**–**1.00)**  **p=0.04** | 1.02 (0.97–1.07)  p=0.52 | 0.97 (0.89–1.05)  p=0.42 | 1.02 (0.87–1.19)  p=0.85 |
| Personal control | 1.00 (0.94–1.06)  p=0.96 | 1.03 (0.95–1.13)  p=0.47 | **1.06 (1.02**–**1.10)**  **p=0.002** | 1.04 (0.99–1.10)  p=0.14 | 1.03 (0.95–1.12)  p=0.45 | 0.98 (0.83–1.15)  p=0.78 |
| Treatment control | 1.04 (0.98–1.12)  p=0.23 | 1.02 (0.92–1.14)  p=0.70 | 0.99 (0.95, 1.02)  p=0.44 | 0.96 (0.90–1.02)  p=0.18 | 0.99 (0.91–1.07)  p=0.72 | 0.91 (0.75–1.11)  p=0.34 |
| Identity | 1.00 (0.93–1.08)  p=0.94 | 0.95 (0.86–1.05)  p=0.30 | **0.94 (0.90**–**0.97)**  **p<0.001** | **1.09 (1.03**–**1.15)**  **p=0.002** | 0.94 (0.86–1.03)  p=0.18 | 0.91 (0.76–1.09)  p=0.33 |
| Concern | 1.05 (0.97–1.13)  p=0.23 | 0.98 (0.91–1.06)  p=0.66 | 0.97 (0.93–1.00)  p=0.08 | 0.96 (0.92–1.00)  p=0.06 | 0.93 (0.86–1.02)  p=0.11 | 1.04 (0.89–1.22)  p=0.60 |
| Coherence | **0.92 (0.87**–**0.98)**  **p=0.008** | 0.95 (0.87–1.04)  p=0.28 | 1.01 (0.98–1.05)  p=0.40 | 0.97 (0.92–1.02)  p=0.23 | 0.96 (0.88–1.03)  p=0.23 | 0.94 (0.79–1.12)  p=0.52 |
| Emotion | 1.04 (0.97–1.11)  p=0.32 | 1.02 (0.93–1.12)  p=0.72 | **0.94 (0.90**–**0.97)**  **p=0.001** | 1.04 (0.98–1.09)  p=0.18 | 0.93 (0.86–1.01)  p=0.08 | 0.92 (0.79–1.08)  p=0.30 |

^1^ AOR: adjusted odds ratios, odds ratios adjusted for background characteristics that were significantly associated with CAS with men among participants in Beijing, including current relationship status, history of confirmed SARS-CoV-2 infection, use of pre-exposure prophylaxis.

^2^ AOR: adjusted odds ratios, odds ratios adjusted for background characteristics that were significantly associated with CAS with men among participants in Hong Kong, including current relationship status, use of any types of HIV testing, use of pre-exposure prophylaxis, testing for other sexually transmitted infections, and use of other HIV-related services.

^3^ Adjusted IRR: incidence rate ratio adjusted for background characteristics that were significantly associated with number of male sex partners with anal intercourse among MSM in Beijing, including age group, current relationship status, employment status, personal income, sexual orientation, use of any types of HIV testing, use of pre-exposure prophylaxis, testing for other sexually transmitted infections, and use of other HIV-related services.

^4^ Adjusted IRR: incidence rate ratio adjusted for background characteristics that were significantly associated with number of male sex partners with anal intercourse among MSM in Hong Kong, including use of any types of HIV testing, use of pre-exposure prophylaxis, testing for other sexually transmitted infections, and use of other HIV-related services.

^5^ AOR: adjusted odds ratios, odds ratios adjusted for background characteristics that were significantly associated with sexualized drug use among participants in Beijing, including employment status, use of any types of HIV testing, use of pre-exposure prophylaxis, and testing for other sexually transmitted infections.

^6^ AOR: adjusted odds ratios, odds ratios adjusted for background characteristics that were significantly associated with sexualized drug use among participants in Hong Kong, including monthly personal income, use of any types of HIV testing, use of pre-exposure prophylaxis, testing for other sexually transmitted infections, and use of other HIV-related services.
